# Mr. Crowfoot, on the Cataract

**Published:** 1802-05

**Authors:** W. Crowfoot

**Affiliations:** Beccles


### *To the Editors of the Medical and Phyſical Journal.*

GENTLEMEN,

I Send you the following Caſe of Cataract, to inſert in the Medical Journal, if you conſider it worthy of publication.

I am, &c.

*Beccles,*

*February* 5, 1802.

W. CROWFOOT.

William Moore, of Long Stratton, a young man, by trade a cooper, applied to me many years ſince, for a defect in his ſight, which within a ſhort time had increaſed ſo much, that he frequently wounded himſelf in the uſual employment of his buſineſs. The cauſe he believed to ariſe from expoſure to a ſevere thunder ſtorm, wherein the lightning was remarkably vivid. Upon examination, a compleat Cataract was obſervable in the right eye; but as the left eye was perfect, and there was reaſon to hope a little time would correct the imperfection he complained of, I was unwilling to adviſe any operation. The gentlemen at the Norwich Hoſpital, with whom he afterwards conſulted, were pleaſed to confirm my opinion, and, as was foretold, he ſoon aſcertained the real ſituation of objects, and was able to follow his accuſtomed occupation without inconvenience. After ſeveral months had elapſed, he was one day ſeized with violent pain in the globe of the diſeaſed eye, attended with fever and delirium. A plentiful bleeding relieved him, but the next day a return of the ſame diſtreſſing ſymptoms came on, when not only general bleeding was repeated, and leeches applied to the temple, but other means recommended in caſes of inflammation were employed, and in a few days he got perfectly well.

Soon after his recovery he made ſimilar miſtakes of objects as he had at firſt, and came very anxiouſly to deſire I would again examine his eyes. It was with great ſurpriſe and admiration I now perceived, that the opacity of the chryſtalline was removed, and except that the iris did not contract ſo readily, he could diſcern objects very diſtinctly with this eye; which, for a ſhort ſpace of time, occaſioned the return of thoſe evils produced by the diſeaſe.

Dr. Murray, a worthy phyſician, at that time in Norwich, who had ſeen him at the hoſpital, examined this patient at my houſe, after his recovery; and it was great ſatisfaction to me, at the time, to have ſuch reſpectable teſtimony to a caſe ſo very extraordinary.

I have ſince heard of two other caſes of Cataract *cured by inflammation*, after unſucceſsful attempts in couching, and one of theſe having happened at a public hoſpital in London, no doubt can remain upon the mind that the fact is well eſtabliſhed.

How far it may be proper or expedient to excite inflammation upon the tunica conjunctiva or cornea, with the deſign of removing a diſeaſe in the chryſtalline, is not for me to determine; but in peruſing a very intereſting caſe of Mr. Ware's in the Philoſophical Tranſactions, I am induced to think the inflammation produced by puncturing the capſule of the chryſtalline, and promoting the abſorption of the opaque humour, may may be an additional reafon to thofe fuggefted by that eminent practitioner, for attempting the cure of blindnefs in cafes of Cataract at an earlier age than was formerly thought advifeable.

